# Chinese Herbal Medicine in the Treatment of Chronic Heart Failure: Three-Stage Study Protocol for a Randomized Controlled Trial

**DOI:** 10.1155/2015/927160

**Published:** 2015-05-18

**Authors:** Liangtao Luo, Jianxin Chen, Shuzhen Guo, Juan Wang, Kuo Gao, Peng Zhang, Chan Chen, Huihui Zhao, Wei Wang

**Affiliations:** ^1^School of Preclinical Medicine, Beijing University of Chinese Medicine, No. 11, Beisanhuan Donglu, Chaoyang District, Beijing 100029, China; ^2^School of Traditional Chinese Medicine, Capital Medical University, No. 10, Youanmenwai, Xitoutiao, Fengtai District, Beijing 100069, China; ^3^Wuhan Hospital of Traditional Chinese Medicine, No. 49, Li Huang Po Lu, Han Kou Jiang An District, Wuhan, Hubei 430014, China; ^4^Hangzhou Xiaoshan District Hospital of Traditional Chinese Medicine, 156 Yu Cai Lu, Xiaoshan District, Zhejiang 311201, China

## Abstract

*Background*. Chinese herbal medicine (CHM) has been used in the treatment of chronic heart failure (CHF) for a long time. Treatment based on syndrome differentiation and the main characteristic of TCM is the fundamental principle of TCM practice. In this study protocol, we have designed a trial to assess the efficacy and safety of CHM on CHF based on syndrome differentiation. *Methods/Design*. This is a three-stage trial of CHM in the treatment of CHF. The first stage is a literature review aiming to explore the common syndromes of CHF. The second is a multicentral, randomized, placebo-controlled trial to evaluate the efficacy and safety of CHM for the treatment of CHF. The third is a multicentral, randomized controlled clinical trial aiming to make cost-effectiveness analysis and evaluate the feasibility, compliance, and universality of CHM on CHF. *Discussion*. This trial will evaluate the efficacy, safety, feasibility, compliance, and universality of CHM on CHF. The expected outcome is to provide evidence-based recommendations for CHM on CHF and develop a prescription of CHM in the treatment of CHF. This trial is registered with NCT01939236 (Stage Two of the whole trial).

## 1. Background

Heart failure, as the final stage of cardiac diseases, is an abnormality of cardiac structure or function. This would lead to the failure of the heart to deliver oxygen at a rate commensurate with the requirements of the metabolizing tissues, despite the normal filling pressures or increased filling pressures [1]. In addition, according to the character of the clinical manifestation, HF is divided into acute and chronic HF. In 2003, a random sample survey of 15,518 urban or rural residents from 35 to 74 years old was made in China: the prevalence rate of heart failure was 0.9%, and according to the result there were approximately 4 million HF targets in China [2]. The diagnosis and treatment of cardiovascular disease (CVD) have developed quickly in the past several decades. The mortality of CVD has fallen down, except for HF. Annual hospital discharge in subjects with a primary diagnosis of HF has been rising steadily since 1975. And now it has exceeded 1 million discharges per year, though they may at last be leveling off in the United States [3, 4]. It can be expected that the incidence of HF in China will be in a significantly increasing trend, and the number of CHF patients will increase in the future.

According to the guideline, the conventional therapeutic approaches in HF management are angiotensin-converting enzyme inhibitors (ACEIs), *β*-adrenergic blockers, and diuretics [5]. However, side effects, such as electrolyte, fluid depletion, and hypotension, are common during the treatment with western medicine [6]. Therefore, CHM, as an alternative medicine, has been considered the treatment of CHF with lower cost and fewer side effects.

In China, CHM has been used in the treatment of diseases for thousands of years. Treatment based on syndrome differentiation is the main characteristic and therapeutic rule of TCM. Syndrome differentiation is the comprehensive analysis of the clinical information gained by the four main diagnostic TCM procedures—observation, listening, interrogation, and pulse-taking, and it is used to guide the choice of treatment by CHM. Many clinical studies [7–9] have shown the efficacy of CHM on CHF. But in these studies, the syndrome of the CHF patients was stationary, and the formulation of CHM was in the form of capsule or injection. They may not completely reflect the efficacy of CHM in the treatment of CHF based on syndrome differentiation. This trial aims to evaluate the efficacy and safety of CHM in the treatment of CHF based on syndrome differentiation.

## 2. Objectives

The objective of this trial is to assess the efficacy and safety of CHM on CHF based on syndrome differentiation and to develop a prescription of CHM in the treatment of CHF which could be generally used in community health service centers.

## 3. Methods 

### 3.1. Design

This is a three-stage, multicentral clinical trial in the treatment of CHF. The first stage is a literature study and aims to explore the common syndromes of CHF. The second stage is a multicentral, randomized controlled clinical trial with double-blinded method and aims to evaluate the efficacy and safety of CHM based on syndrome differentiation. The third stage is a multicentral, randomized controlled clinical trial and aims to make cost-effectiveness analysis and evaluate the feasibility, compliance, and universality of CHM on CHF based on syndrome differentiation.

#### 3.1.1. Stage One

**Figure 1:**
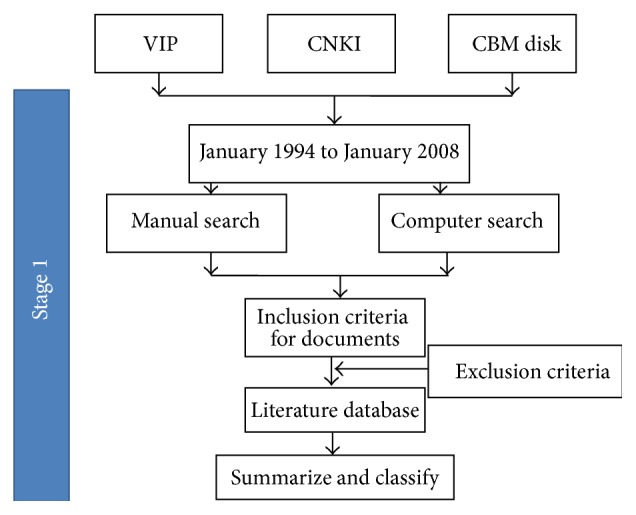
The flowchart of Stage One trial design.

The first stage was to analyze the most common syndromes of CHF. A flowchart of Stage One is shown in Figure 1.

*Inclusion Criteria for the Literature*. The literatures included should focus on clinical research of CHM or integrative medicine on CHF.

The literatures should have integral data with exact data information about CHF syndromes. 

*Exclusion Criteria for the Literature*. If the content of articles was identical and the study was identified to be the same one, we select one article with the most complete information:

 literature reviews,
 case reports,
 export advice,
 the literature focusing on the analysis of pathogenesis or the syndromes substantive,
 the source of the literature being unknown or the actual situation being clearly incompatible with the clinical literature.

*Search Range for Articles*. We searched the China National Knowledge Infrastructure Database (CNKI), the China Biology Medicine Database (CBM), and the Chinese Science and Technology Periodical Database (VIP) from January 1994 to January 2008. Databases search strategies are shown in Table 1. 

*Search Methods*. The literature search work was carried out by 2 students independently in the same search terms. After all work was accomplished, we checked the search result and resolved the differences through discussion. If the disagreements still could not be resolved, we invited related experts for identification. 

*Selecting the Content of Related Literatures*. We selected records of CHF and syndromes and related information. 

*The Processing Methods of Selected Records*. We picked out TCM syndromes, symptoms, and signs in records and attached the related information, such as paper title, journal name, issue number, page count, and author.

All data were input into database established with Epidata 3.0. The frequency of common syndromes and symptoms was analyzed. We encoded these common syndromes and symptoms and regulated the related TCM terms. We unified the TCM names or aliases with the same meaning but different expression into uniform names. 

*Summarizing and Classifying the Findings of Literatures*. We summarized the clinical characteristics and distribution of CHF common syndromes from the results of searched records. There were 1432 literatures with full text search in three databases. According to inclusion and exclusion criteria, we selected 176 literatures from the three literature databases. According to Clinical Terminology of Diagnosis and Treatment for Traditional Chinese Medicine—Syndrome Section and Pharmacy Terminology of Traditional Chinese Medicine, with the suggestion of experts, we standardized all symptoms and syndromes of CHF. There were twelve symptoms with more than 100 repetitions after standardization, which were shown in Table 2. The top six symptoms are yang deficiency, qi deficiency, blood stasis, water retention, yin deficiency, and turbid phlegm, which were shown in Table 3 [10]. Combined with the literature review and experts' suggestions, we decided to make qi deficiency, blood stasis, yang deficiency, and water retention as objects for the further trial of Stages Two and Three.

#### 3.1.2. Stage Two

**Figure 2:**
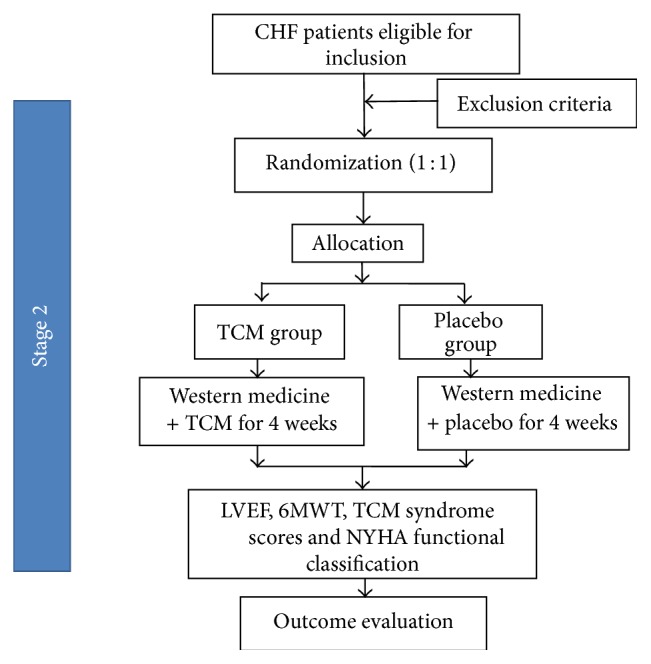
The flowchart of Stage Two trial design.

The second stage of this trial was a multicentral, randomized clinical trial with placebo-controlled and double-blind methods.  Figure 2 shows the trial flowchart. 

*Diagnostic Criteria*

*Diagnostic Criteria for CHF*. Diagnostic criteria were as follows: (1) diagnostic criteria of CHF:* 2007 China Guideline for the Diagnosis and Treatment of CHF* [5]; (2) heart function standard:* The Criteria for Diagnosis and Treatment of Heart Disease *first published by the New York Heart Association (NYHA) [11]. 

*Diagnostic Criteria for TCM Syndrome Differentiation*. Diagnostic criteria were as follows: TCM differentiation standard: according to the* Guiding Principles for the Clinical Study of New Drugs in Traditional Chinese Medicine* released in 2002 [12]. 

*Inclusion Criteria for Participants*. Patients who accorded with diagnostic criteria were potentially eligible for the study if they met the following criteria.

- The primary heart disease is CHD (with diagnosis for CHD confirmed by coronary angiography, coronary computed tomography, history of acute myocardial infarction, limb-salvage Q wave for electrocardiogram (ECG), ECG test, radionuclide examination support, etc.). The included patients should have no history of hypertension or taking antihypertensive drugs, with a blood pressure under 160/100 mmHg.
- With a history of CHD, the following symptoms and signs were observed: difficult breathing, fatigue, fluid retention (edema), left ventricular enlargement, systolic volume of left ventricular increase with LVEF ≤ 40%, and NYHA functional classification II or III.
- Male or female patients should be between 40 and 75 years old.

If there was any violation of those criteria, the subject could not participate in this research.

*Exclusion Criteria for Participants*. Patients would be excluded if they met one of the following criteria.

- Patients with one of the following diseases: (1) acute valvular heart disease; (2) pericardial disease; (3) cardiomyopathy; (4) congenital heart disease; (5) acute myocardial infarction (AMI) within four weeks; (6) cardiac shock; (7) acute myocarditis or serious arrhythmia with variation in hemodynamics.
- Patients who suffer from pulmonary artery hypertension caused by cor pulmonale, pulmonary embolism, or stroke within a half year.
- Patients who suffer from serious hepatic insufficiency (the index of liver function being 2 times the normal one), renal insufficiency (Ccr > 20%, Scr > 3 mg/dL, or 265 *μ*mol/L), diseases of blood system, malignant tumor, diabetes mellitus with serious complications, hyperthyrea, or hypothyrea.
- Patients who suffer from infection, fever, or patients meeting one of the following criteria: the numeration of leukocyte being more than 10 × 10^9^/L, the percentage of neutrophil granulocyte being more than 85%; patchy shadows in X-ray of chest.
- Pregnant women and women in lactation.
- Patients with mental disorders or related infections.
- Patients who took part in other trials within two months before the present study.

*Sample Size*. The sample size calculation is based on prophase clinical study (31 cases, 13 in CHM group and 18 in placebo group), standard deviation of experimental group (*S*_*e*_) = 25.84718, standard deviation of control group (*S*_*c*_) = 32.54499, mean of experimental group (${\bar{x}}_{e}$) = 39.07692, mean of control group (${\bar{x}}_{c}$) = 32.54499, and the number of control group (*c*) = 1. The following formula is used to calculate the sample size:

(1) $$\begin{array}{c} n=\frac{{\left(\upsilon_{\alpha }+\upsilon_{\beta }\right)}^{2}\left(1+1/c\right)\sigma^{2}}{\delta^{2}}\\ \sigma^{2}=S^{2}=\frac{S_{e}^{2}+cS_{c}^{2}}{1+c}\delta^{2}={\left|{\bar{x}}_{e}-{\bar{x}}_{c}\right|}^{2}. \end{array}$$

The estimated number of subjects in each group was 98.18012, and 196.36024 subjects are needed in two groups. The number was increased to 215.18012 assuming a maximum dropout rate of less than or equal to 10%, and a total of 220 subjects were included (110 subjects in each group).

*Randomization*. A total number of 220 participants were randomly assigned with a ratio of 1 : 1 to CHM or placebo group according to random number sequence. Stratified blocked randomization was taken to insure the balance of two groups.

*Blinding*. In this stage, the double-blind design was used. Drugs for CHM and placebo groups were prepared and provided by Beijing Kangren Tang Pharmaceutical Co., Ltd. (Beijing, China). In CHM group, drugs of four syndromes (qi deficiency, blood stasis, water retention, and yang deficiency) were prepared, while the placebo was provided in the same character, smell, and weight as the drugs for CHM group. Drugs of each syndrome were prepared as granules without decoction. The CHM drugs and placebo were numbered according to a random figure table which was blinded to both the doctors and the patients. Participants were provided with CHM or placebo based on their syndrome differentiation at the beginning and two weeks later. They took CHM or placebo twice per day for four weeks.

*Setting*. In this stage, participants were admitted into seven hospitals of four provinces in China: (1) Dongfang Hospital Affiliated to Beijing University of Chinese Medicine; (2) The Affiliated Hospital to Changchun University of TCM; (3) Guang'anmen Hospital Affiliated to China Academy of Chinese Medicine Sciences; (4) Hubei Provincial Hospital of TCM; (5) Wuhan Hospital of TCM; (6) Yichang Hospital of TCM; (7) Zhengzhou Hospital of TCM.

*Interventions*. According to the guideline for chronic heart failure [5], standardized western medicine treatment, such as angiotensin-converting enzyme inhibitors (ACEIs) or angiotensin-receptor blockers (ARBs), *β*-blockers, and diuretics, could be used for patients in CHM and placebo groups as basic therapy. The dosages of all medicines used are following the guideline for CHF [5].

The recipe for qi deficiency was composed of* Astragalus membranaceus* (Fisch.) Bge. var.* mongholicus *(Bge.) Hsiao (huangqi, 60 g) and* Codonopsis pilosula *(Franch.) Nannf. (dangshen, 15 g).

The recipe for blood stasis was composed of* Salvia miltiorrhiza *Bge. (danshen, 15 g),* Paeonia lactiflora *Pall. (chishao, 15 g),* Prunus davidiana* (Carr.) Franch. (taoren, 10 g), and* Carthamus tinctorius* L (honghua, 10 g).

The recipe for water retention was composed of* Alisma orientalis *(Sam.) Juzep. (zexie, 10 g),* Polyporus umbellatus* (Pers.) Fries (zhuling, 15 g),* Plantago asiatica* L. (cheqianzi, 10 g), and* Descurainia sophia *(L.) Webb. ex Prantl (tinglizi, 10 g).

The recipe for yang deficiency was composed of* Cinnamomum cassia *Presl (rougui, 4.5 g) and* Aconitum carmichaelii *Debx. (zhifuzi, 10 g).

Table 6 lists the names of the CHM drugs in Chinese and English. All CHM herbs were verified by high-performance liquid chromatography (HPLC).

Patients in placebo group received placebo which was the same in shape, size, taste, weight, and package as CHM granules. CHM drugs and placebo were prepared as granules. Granules quality met internal control standards of Beijing Kangren Tang Pharmaceutical Co., Ltd. (Beijing, China), and complied with GMP (Good Manufacturing Practice) standards. Patients were treated with CHM or placebo granules twice per day for four weeks based on syndrome differentiation.

Participants were prohibited from taking other Chinese medicine during the treatment period.

The drugs for the treatment of hypertension (HBP), diabetes mellitus, dyslipidemia, and other diseases could be used reasonably. And the reason, name, and dosage should be recorded in detail.

*Outcome Measurements*. The primary outcome measure was left ventricular ejection fraction (LVEF). Secondary outcome measurements were 6-minute walk test (6MWT), traditional Chinese medicine syndrome scores, and New York Heart Association (NYHA) functional classification. Side effects were to be monitored during the trial period. Many biological indicators (blood routine examination, liver function, kidney function, and ECG) were tested before and after the treatment. The timeline of participants through Stage Two is shown in Table 4.

*Statistical Analysis*. Data were analyzed on the Intention-to-Treat (ITT) analysis and the per-protocol set (PPS) for coherence. The basic characteristics were compared with independent samples *t*-test for continuous variables and chi-square analyses for categorical variables. Repeated-measure ANOVA was used for the evaluation of the primary and secondary outcomes. The measurement data were expressed as mean ± standard deviation for us to check the data of all groups with normal test and homogeneity of variance test. Results were shown with 95% confidence intervals (CIs). The level of significance was set at 0.05. If *P* < 0.05, there were statistical differences. All tests were 2-tailed.

#### 3.1.3. Stage Three

**Figure 3:**
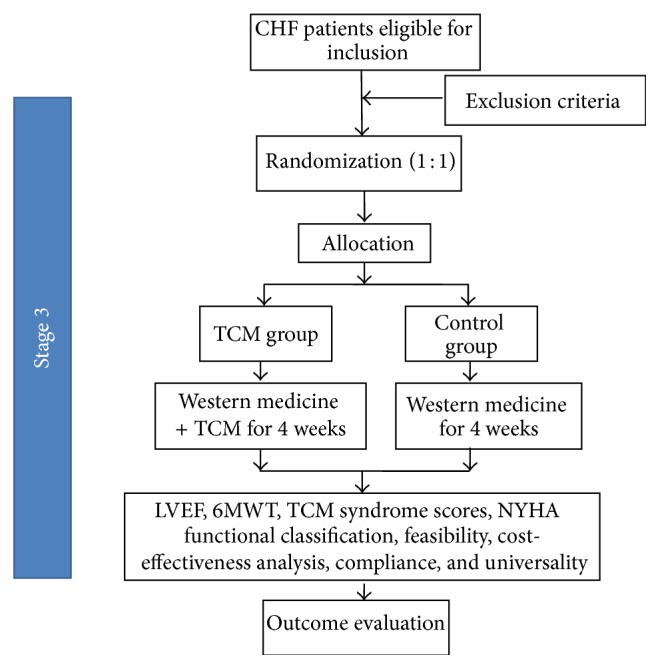
The flowchart of Stage Three trial design.

If the efficacy of CHM on CHF is better than placebo in Stage Two, we will conduct Stage Three to evaluate the feasibility, compliance, and universality and make cost-effectiveness analysis of CHM on CHF based on syndrome differentiation. A flowchart of the third stage is shown in Figure 3.

*Diagnostic Criteria*

*Diagnostic Criteria for CHF*. Diagnostic criteria are the same as those of Stage Two.

*Diagnostic Criteria for TCM Syndrome Differentiation*. Diagnostic criteria were the same as those of Stage Two.

*Inclusion Criteria for Participants*. Patients who accorded with diagnostic criteria were potentially eligible for the study if they met the following criteria.

- The primary heart disease is CHD (with diagnosis for CHD confirmed by coronary angiography, coronary computed tomography, history of acute myocardial infarction, limb-salvage Q wave for electrocardiogram (ECG), ECG test, radionuclide examination support, etc.). The included patients also had no history of hypertension or taking antihypertensive drugs, with a blood pressure under 160/100 mmHg.
- With a history of CHD, the following symptoms and signs were observed: difficult breathing, fatigue, and fluid retention (edema), with left ventricular enlargement, along with increasing of left ventricular end systolic volume and LVEF ≤ 50%, with NYHA cardiac function II or III.
- Male or female patients should be between 40 and 75 years old.

If there is any violation of those criteria, the subject could not participate into this research.

*Exclusion Criteria for Participants*. Exclusion criteria were the same as those in Stage Two.

*Sample Size*. This design in this stage is to show the superiority of TCM based on syndrome differentiation. The ratio in this stage is 1 : 1. The sample size calculation is based on LVEF of Stage Two. To calculate the sample size, we will use the following formula (*δ* = 2):

(2) $$\begin{array}{c} n=\frac{\left({\left(Z_{1-a/2}+Z_{1-\beta }\right)}^{2}*\left(\sigma_{1}^{2}+\sigma_{2}^{2}\right)\right)}{{\left(\varepsilon -\delta \right)}^{2}}. \end{array}$$

And the maximum dropout is 10%.

*Randomization*. All participants in this stage will be randomly distributed to CHM or placebo group according to random number sequence.

*Setting*. In this stage, five or more centers will take part in the trial.

*Interventions*. Besides standardized western medicine treatment, participants in CHM group will receive CHM based on syndrome differentiation. Control Group will receive standardized western medicine only.

CHM granules will be prepared as in Stage Two.

*Outcome Measurements*. In this stage, the primary outcome measurement is LVEF. Secondary outcome measurements are 6MWT, TCM syndrome scores, and NYHA functional classification. Meanwhile, the feasibility, cost-effectiveness analysis, compliance, and universality will be evaluated or performed. Feasibility and universality investigation of this research will be executed by the questionnaire of doctors and patients; compliance evaluation will be executed by the ratio between the actual amount of medication and the supposed amount in CHM group; cost effectiveness will be conducted by the ratio between the different improved condition of LVEF and treatment costs. Side effects will be monitored during the trial period. Biological indicators (blood routine examination, liver function, kidney function, and ECG) will be tested before and after the treatment. The timeline of participants through Stage Three is shown in Table 5.

*Statistical Analysis*. Data will be analyzed on the Intention-to-Treat (ITT) analysis and the per-protocol set (PPS) for coherence. The basic characteristics will be compared with independent samples *t*-test for continuous variables and chi-square analyses for categorical variables. The measurement data will be expressed as mean ± standard deviation for us to check the data of all groups with normal test and variance homogeneity test. The changes in measurement data between baseline and the assessment for four weeks will be performed with independent samples *t*-test. The categorical variables will be compared with chi-square analyses. Safety analysis will be conducted. Results will be shown with 95% confidence intervals (CIs). The level of significance will be set at 0.05. If *P* < 0.05, there will be statistical differences. All tests will be 2-tailed.

### 3.2. Ethical Issue of Stages Two and Three

The trial is conducted according to the guidelines of the Declaration of Helsinki and is approved by the Ethics Committee of Dongfang Hospital Affiliated to Beijing University of Chinese Medicine (number 201002102). All participants have been asked to provide informed consent before participating in the trial.

### 3.3. Quality Control of Stages Two and Three

Research workbook will be formulated. Before the beginning of this stage, all investigators will be trained and tested according to conformance standard. Drugs in this trial will be identified by pharmaceutical manufacturing sector and prepared in accordance with national technical standards procedures. Drugs will be marked only for clinical trial, which cannot be sold. The investigators in clinical centers will take charge of the usage of drugs. The usage record of drugs should include the following information: quantity, shipment, delivery, acceptance, distribution, recycling, and destruction.

In order to achieve the same detection methods, the physicians for 6MWT evaluation and echocardiography will be trained together. Clinical research associates will test the research progress of all clinical centers.

## 4. Discussion

CHF is a major public health problem all over the world. Western medicine has been used widely but has many common side effects [6]. In China, the integrated traditional and western medicine has been used for the treatment of CHF for a long time, and its efficacy has been seen in many clinical trials [13]. However, there is insufficient evidence to support the efficacy of CHM based on syndrome differentiation.

In TCM, patients are treated with different therapies based on their syndrome differentiations. Therefore, the key factor of this trial is to find out the common syndromes of CHF patients. At the first stage, we searched the most common syndromes.

This is the first randomized clinical trial on CHF with CHM based on syndrome differentiation. The aim of this trial is to develop a prescription of CHM in the treatment of CHF which could be used in community health service centers. So in Stage Two, we tested the efficacy and safety of CHM on CHF with a double-blind, randomized, placebo-controlled clinical trial. In Stage Three, we will test the feasibility, cost-effectiveness analysis, compliance, and universality of those CHMs, which have showed positive sign in efficacy and safety in Stage Two. And finally, according to the result of Stages Two and Three, we will decide whether this CHM treatment proposal for CHF should be generalized in community health service centers or not in the future.

In conclusion, we want to evaluate the efficacy, safety, feasibility, cost-effectiveness analysis, and universality of CHM treatment on CHF based on syndrome differentiation with three stages. The achievement of this trial will provide evidence-based data for CHM, which is helpful for the application of CHM on CHF.

*Trial Status.* Stages One and Two of this trial have been accomplished. Stage Three will be started in November 2013 and will be completed in September 2014.

## Figures and Tables

**Table 1:** Databases search strategies.

Databases	Search terms
CNKI	[(Mesh terms = heart failure) OR (Title = chronic heart failure) OR (Keywords = chronic heart failure)] AND [(Mesh terms = syndrome) OR (Keywords = syndrome)]

CBM	(Mesh terms = chronic heart failure OR Mesh terms = CHF OR Mesh terms = chronic congestive heart failure OR Mesh terms = chronic cardiac insufficiency) and ((Mesh terms = syndrome) or (Mesh terms = traditional Chinese medicine))

VIP	((Title or keywords: chronic cardiac insufficiency) OR (Title or keywords: CHF) OR (Title or keywords: chronic heart failure) OR (Title or keywords: chronic congestive heart failure)) AND (Title or keywords: syndrome) AND (Title or keywords: symptom) AND (Medicine and health) AND (All Periodicals) AND (Year: 1994–2008)

**Table 2:** The distribution of symptoms in 176 literatures.

Symptom	Frequency	Rate (%)
Palpitation	233	5.6
Shortness of breath	160	3.9
Edema	159	3.8
Lassitude	153	3.7
Thread pulse	146	3.5
Dyspnea	126	3.0
Wheeze	117	2.8
Pale tongue	111	2.7
Intermittent pulse	110	2.7
Knotted pulse	110	2.7
Oliguria	101	2.4
Sweating	100	2.4

**Table 3:** The distribution of syndromes in 176 literatures.

Syndrome	Frequency	Rate (%)
Yang deficiency	6489	18.57
Qi deficiency	6377	18.25
Blood stasis	5731	16.40
Water retention	3445	9.86
Phlegm syndrome	973	2.78

**Table 4:** The timeline of participants' course through Stage Two.

Items	Baseline	Treatment	
	Visit 1	Visit 2	Visit 3
	0 weeks	2 weeks	4 weeks
Inclusion/exclusion	•		
Informed consent	•		
Grouping	•		
Medical history collection	•		
General information	•		
Medication history	•		
Physical examination	•		
Complicating diseases	•		
Drug release	•	•	
TCM syndrome scores	•	•	•
NYHA functional classification	•	•	•
6-minute walk test	•	•	•
LVEF	•	•	•
X-ray	•		
Routine urine test	•		•
Liver function test	•		•
Renal function test	•		•
Electrolytes	•		•
Electrocardiogram	•	•	•
Side effect		•	•

**Table 5:** The timeline of participants' course through Stage Three.

Items	Baseline	Treatment	
	Visit 1	Visit 2	Visit 3
	0 weeks	2 weeks	4 weeks
Inclusion/exclusion	•		
Informed consent	•		
Grouping	•		
Medical history collection	•		
General information	•		
Medication history	•		
Physical examination	•		
Complicating diseases	•		
Drug release	•	•	
TCM syndrome scores	•		•
NYHA functional classification	•		•
6-minute walk test	•		•
LVEF	•		•
X-ray	•		
Routine urine test	•		•
Liver function test	•		•
Renal function test	•		•
Electrolytes	•		•
Electrocardiogram	•		•
Feasibility			•
Cost-effectiveness analysis			•
Compliance			•
Universality			•
Side effect		•	•

**Table 6:** List of CHMs used in the study.

Syndrome differentiation	English translation of CHM	Chinese script
Qi deficiency	Huangqi	*黄芪*
	Dangshen	*党参*

Blood stasis	Danshen	*丹参*
	Chishao	*赤芍*
	Taoren	*桃仁*
	Honghua	*红花*

Water retention	Zexie	*泽泻*
	Zhuling	*猪苓*
	Cheqianzi	*车前*子
	Tinglizi	*葶苈*子

Yang deficiency	Zhifuzi	*制附*子
	Rougui	*肉桂*

## Abbreviations

6MWT: 6-minute walk test
ACEI: Angiotensin-converting enzyme inhibitors
AMI: Acute myocardial infarction
CBM: China Biology Medicine Database
CHD: Coronary heart disease
CHF: Chronic heart failure
CHM: Chinese herbal medicine
CIs: Confidence intervals
CNKI: China National Knowledge Infrastructure Database
CVD: Cardiovascular disease
ECG: Electrocardiogram
GMP: Good Manufacturing Practice
HBP: Hypertension
ITT: Intention-to-Treat
LVEF: Left ventricular ejection fraction
NYHA: New York Heart Association
PPS: Per-protocol set
RCT: Randomized controlled trial
TCM: Traditional Chinese medicine
VIP: Chinese Science and Technology Periodical Database.
